# Brief Report: Updated Efficacy and Safety Data From an Integrated Analysis of Entrectinib in Locally Advanced/Metastatic *ROS1* Fusion-Positive Non–Small-Cell Lung Cancer

**DOI:** 10.1016/j.cllc.2023.12.001

**Published:** 2024-03

**Authors:** Yun Fan, Alexander Drilon, Chao-Hua Chiu, Herbert H.F. Loong, Salvatore Siena, Maciej Krzakowski, Rafal Dziadziuszko, Harald Zeuner, Cloris Xue, Matthew G. Krebs

**Affiliations:** 1 Department of Thoracic Medical Oncology, The Cancer Hospital of the University of Chinese Academy of Sciences (Zhejiang Cancer Hospital), Hangzhou, China; 2 Memorial Sloan Kettering Cancer Center, and Weill Cornell Medical College, New York, NY; 3 Department of Chest Medicine, Taipei Veterans General Hospital, Taipei, Taiwan; 4 Taipei Cancer Center and Taipei Medical University Hospital, Taipei Medical University, Taipei, Taiwan; 5 Department of Clinical Oncology, The Chinese University of Hong Kong, Hong Kong SAR, Hong Kong; 6 Niguarda Cancer Center, Grande Ospedale Metropolitano Niguarda, Milan, Italy; 7 Department of Oncology and Hemato-Oncology, Università degli Studi di Milano, Milan, Italy; 8 Lung Cancer and Thoracic Cancer Department, Maria Sklodowska-Curie National Research Institute of Oncology, Warsaw, Poland; 9 Department of Oncology and Radiotherapy and Early Clinical Trials Center, Medical University of Gdansk, Gdansk, Poland; 10 F. Hoffmann-La Roche Ltd, Basel, Switzerland; 11 F. Hoffmann-La Roche Ltd, Mississauga, Canada; 12 Division of Cancer Sciences, Faculty of Biology, Medicine and Health, The University of Manchester, and The Christie NHS Foundation Trust, Manchester Academic Health Science Centre, Manchester, United Kingdom

**Keywords:** First-line treatment, Intracranial efficacy, NSCLC, Tyrosine kinase inhibitor

## Abstract

• Genetic alterations in *ROS1* can lead to the expression of oncogenic fusion proteins in multiple tumor types, including in 1% to 2% of non–small-cell lung cancer (NSCLC) cases. Approximately 40% of patients with *ROS1* fusion-positive NSCLC have baseline central nervous system (CNS) metastases, indicating the need for a treatment with CNS activity. Entrectinib, a potent ROS1 tyrosine kinase inhibitor with activity in the CNS, has previously demonstrated overall and intracranial efficacy, and a manageable safety profile, in patients with *ROS1* fusion-positive NSCLC.
• In this updated analysis with 4 additional patients and longer follow-up, the objective response rate (ORR) in the efficacy-evaluable population (N = 172) was 67%; median duration of response (DoR) was 20.4 months, and median progression-free survival was 16.8 months. In 51 patients with baseline CNS metastases, intracranial ORR was 49% and median intracranial DoR was 12.9 months. In a subgroup analysis in patients who had not received any prior systemic therapy in the metastatic setting, ORR was similar to that in the efficacy-evaluable population, but median DoR was numerically longer at 35.6 months. Most treatment-related adverse events were grade 1 to 2 and nonserious.
• These data reinforce previous findings on the use of entrectinib for the treatment of patients with *ROS1* fusion-positive NSCLC, and support current guidelines that recommend entrectinib as a first-line treatment option for these patients, including those with baseline CNS metastases.

## Introduction

*ROS* proto-oncogene 1 (*ROS1*) rearrangements, found in 1% to 2% of patients with non–small-cell lung cancer (NSCLC), can result in the expression of oncogenic fusion proteins in different tumor types.^1^^,^^2^ Approximately 40% of patients with *ROS1* fusion-positive NSCLC have central nervous system (CNS) metastases at diagnosis of advanced disease.^3^^,^^4^^,^^5^^,^^6^

Entrectinib is a potent, CNS-active, ROS1 tyrosine kinase inhibitor (TKI) with demonstrated efficacy in *ROS1* fusion-positive NSCLC.^7^^,^^8^^,^^9^ In an integrated analysis of 3 phase I/II trials (ALKA-372-001: EudraCT 2012–000148–88; STARTRK-1: NCT02097810; STARTRK-2: NCT02568267), entrectinib yielded an objective response rate (ORR) of 68%, median duration of response (DoR) of 20.5 months and median progression-free survival (PFS) of 15.7 months, and was well tolerated in patients with locally advanced/metastatic *ROS1* fusion-positive NSCLC (N = 168).^9^ Entrectinib also showed durable intracranial responses: intracranial ORR was 52% and median intracranial DoR was 12.9 months, in patients with measurable and nonmeasurable baseline CNS metastases by blinded independent central review (BICR; n = 48).^9^

We present updated efficacy and safety data from the integrated analysis, with 4 more patients and longer follow-up (median follow-up of 37.8 months vs. 29.1 months previously).^9^ We also present the first report of an exploratory subgroup analysis of entrectinib in patients with *ROS1* fusion-positive NSCLC who had not received any prior systemic therapy in the metastatic setting (first-line population).

## Methods

### Study Design and Patients

Full details of the entrectinib studies in the integrated analysis, including definition of study endpoints, have been published previously (protocols available online).^7^^,^^8^^,^^9^ Briefly, patients aged ≥18 years with locally advanced/metastatic ROS1 TKI-naïve *ROS1* fusion-positive NSCLC, were enrolled in 1 of 3 single-arm trials (ALKA-372-001, STARTRK-1, and STARTRK-2). Patients received entrectinib 600 mg/day orally, until documented disease progression (PD), unacceptable toxicity, or consent withdrawal. The efficacy-evaluable population comprised all patients who received ≥1 entrectinib dose, had an Eastern Cooperative Oncology Group performance status (ECOG PS) 0 to 2, measurable disease at baseline, and ≥12 months follow-up from the first post-treatment initiation tumor assessment; patients who discontinued from the study or died before completing 12 months of follow-up from first post-treatment tumor assessment were included in the analysis. Patients with asymptomatic or previously treated, controlled CNS metastases were also eligible. The first-line population comprised efficacy-evaluable patients who had not received any prior systemic therapy in the metastatic setting. Tumor assessments (by BICR per RECIST v1.1) were performed at the end of cycle 1 (week 4), and then every 8 weeks. Brain scans were undertaken at every tumor assessment in patients with investigator-assessed baseline CNS metastases and only when clinically indicated or when scans were routinely offered in clinical practice in patients without baseline CNS metastases.

The safety-evaluable population comprised all patients who received ≥1 dose of entrectinib. Details on safety assessments and dose reductions can be found in the Supplement.

### Endpoints

Coprimary endpoints were confirmed ORR and DoR, both by BICR. Secondary endpoints were PFS (by BICR), OS, intracranial ORR (per RECIST v1.1), intracranial DoR, intracranial PFS, and safety. Intracranial efficacy was assessed on CNS lesions (measurable and nonmeasurable). The enrolment cut-off for this analysis was July 2, 2020 and the data cut-off was August 2, 2021.

All studies included in this analysis were conducted in accordance with the principles of the Declaration of Helsinki and Good Clinical Practice Guidelines. Written informed consent was obtained from all patients. Protocols for all studies were approved by relevant institutional review boards and ethics committees.

Details on statistical analyses are provided in the Supplement.

## Results

### Baseline Demographics and Disease Characteristics

The efficacy-evaluable population comprised 172 patients, of whom 67 had received no prior systemic therapy in the metastatic setting (ie, the first-line population). Median survival follow-up was 37.8 months (95% confidence interval [CI]: 35.9-41.4) for the efficacy-evaluable and 41.4 months (95% CI: 35.9-43.4) for the first-line population. Baseline demographics and disease characteristics were similar across the efficacy-evaluable and first-line populations (Supplemental Table 1). Forty patients (23%) in the efficacy-evaluable population had received ≥2 prior lines of treatment for metastatic disease. Baseline CNS metastases assessed by the investigator were present in 35% of patients (n = 60) in the efficacy-evaluable and 39% (n = 26) of patients in the first-line population; of these, 45% (n = 27) and 42% (n = 11), respectively, had received prior radiotherapy to the brain. In total, 9% of patients in the efficacy-evaluable and 8% of patients in the first-line population had an ECOG PS of 2.

### Entrectinib in All Patients With ROS1 Fusion-Positive NSCLC

**Figure 1:**
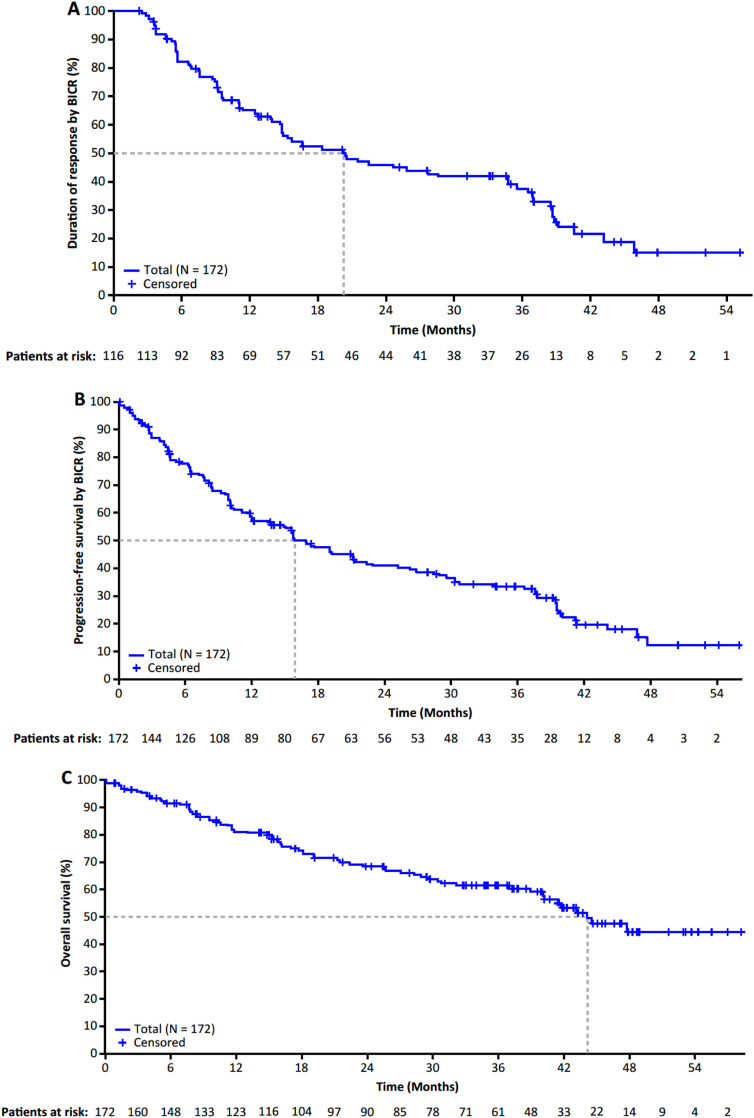
Time-to-event analysis for (A) duration of response, (B) progression-free survival, and (C) overall survival with entrectinib in patients with *ROS1* fusion-positive NSCLC who were ROS1 TKI–naïve (efficacy-evaluable population). Abbreviations: BICR = blinded independent central review; CI = confidence interval; NSCLC = non–small-cell lung cancer; *ROS1* = *ROS* proto-oncogene 1; TKI = tyrosine kinase inhibitor.

ORR in the efficacy-evaluable population (N = 172) was 67% (n = 116; 95% CI: 59.9-74.4) and was similar in patients with and without baseline CNS metastases (Table 1). In the overall efficacy-evaluable population, patients demonstrated durable responses with a median DoR of 20.4 months (95% CI: 14.8-34.8; Figure 1A), while patients with and without baseline CNS metastases achieved a median DoR of 14.6 and 28.6 months, respectively (Table 1). Entrectinib also demonstrated prolonged survival; median PFS was 16.8 months (95% CI: 12.2-22.4) and OS remains immature in the efficacy-evaluable population (Figure 1B and C; Table 1).

**Table 1:** Overall Efficacy in All Patients With *ROS1* Fusion–Positive NSCLC Who Were ROS1 TKI–Naïve (Efficacy-Evaluable Population) and in Patients With *ROS1* Fusion-Positive NSCLC Who Received Entrectinib as First-Line Treatment, According to the Presence/Absence of Measurable and Nonmeasurable Baseline CNS Metastases by the Investigator

Efficacy Parameter	Efficacy-Evaluable Population (N = 172)	Baseline CNS Metastases^a^ (n = 60)	No Baseline CNS Metastases^a^ (n = 112)	First-Line Population^b^ (n = 67)	Baseline CNS Metastases^a^ (n = 26)	No Baseline CNS Metastases^a^ (n = 41)
Objective response, n (%) (95% CI)	116 (67.4) (59.9-74.4)	38 (63.3) (49.9-75.4)	78 (69.6) (60.2-78.0)	46 (68.7) (56.2-79.4)	17 (65.4) (44.3-82.8)	29 (70.7) (54.5-83.9)
Best overall response, n (%)						
Complete response	23 (13.4)	4 (6.7)	19 (17.0)	10 (14.9)	3 (11.5)	7 (17.1)
Partial response	93 (54.1)	34 (56.7)	59 (52.7)	36 (53.7)	14 (53.8)	22 (53.7)
Stable disease	16 (9.3)	6 (10.0)	10 (8.9)	7 (10.4)	5 (19.2)	2 (4.9)
Progressive disease	16 (9.3)	8 (13.3)	8 (7.1)	5 (7.5)	1 (3.8)	4 (9.8)
Non-CR/non-PD	10 (5.8)	2 (3.3)	8 (7.1)	6 (9.0)	1 (3.8)	5 (12.2)
Missing or unevaluable^c^	14 (8.1)	6 (10.0)	8 (7.1)	3 (4.5)	2 (7.7)	1 (2.4)
Median DoR, months (95% CI)	20.4 (14.8-34.8)	14.6 (11.0-20.4)	28.6 (14.9-38.6)	35.6 (13.9-38.8)	16.5 (9.2-35.6)	40.5 (13.9-NE)
Patients with event, n (%)	76 (65.5)	27 (71.1)	49 (62.8)	27 (58.7)	14 (82.4)	13 (44.8)
12-month durable response, % (95% CI)	65.0 (56.1-73.9)	60.0 (43.7-76.3)	67.2 (56.7-77.8)	64.2 (50.1-78.3)	58.8 (35.4-82.2)	67.7 (50.3-85.1)
18-month durable response, % (95% CI)	52.2 (42.8-61.7)	42.6 (25.3-60.0)	56.2 (45.0-67.5)	56.5 (41.7-71.4)	44.1 (19.2-69.0)	63.7 (45.6-81.8)
Median PFS, months (95% CI)	16.8 (12.2-22.4)	11.8 (7.2-15.7)	25.2 (15.7-36.6)	17.7 (11.8-39.4)	11.9 (7.7-21.1)	37.7 (14.8-NE)
Patients with event, n (%)	118 (68.6)	46 (76.7)	72 (64.3)	43 (64.2)	22 (84.6)	21 (51.2)
12-month durable response, % (95% CI)	57.7 (50.0-65.3)	45.6 (32.6-58.7)	64.1 (54.9-73.2)	58.3 (46.2-70.3)	45.8 (26.6-65.1)	66.6 (51.7-81.5)
18-month durable response, % (95% CI)	47.3 (39.5-55.2)	32.9 (20.1-45.7)	54.8 (45.2-64.5)	49.5 (37.1-62.0)	32.4 (13.7-51.1)	60.8 (45.2-76.4)
Median OS, months (95% CI)	44.1 (40.1-NE)	28.3 (17.0-44.6)	NE (41.8-NE)	47.7 (43.2-NE)	43.2 (16.1-NE)	NE (NE)
Patients with event, n (%)	67 (39.0)	31 (51.7)	36 (32.1)	23 (34.3)	14 (53.8)	9 (22.0)
12-month durable response, % (95% CI)	81.3 (75.2-87.3)	74.1 (62.3-85.9)	84.9 (78.1-91.7)	82.7 (73.4-92.0)	76.3 (59.6-92.9)	87.1 (76.4-97.7)
18-month durable response, % (95% CI)	74.3 (67.4-81.2)	63.1 (49.7-76.5)	79.8 (72.0-87.5)	75.8 (65.1-86.5)	68.0 (49.6-86.3)	81.1 (68.5-93.8)

Abbreviations: BICR = blinded independent central review; CI = confidence interval; CNS = central nervous system; CR = complete response; DoR =duration of response; NE = not estimable; NSCLC = non–small-cell lung cancer; OS = overall survival; PD = progressive disease; PFS = progression-free survival; RECIST = Response Evaluation Criteria in Solid Tumors; *ROS1* = *ROS* proto-oncogene 1; TKI = tyrosine kinase inhibitor.

Objective response rate, duration of response, and progression-free survival by BICR per RECIST v1.1.

a Baseline CNS metastases as assessed by the investigator. ^b^ Patients who had not received any prior lines of systemic therapy in the metastatic setting; exploratory analysis. ^c^ Missing or unevaluable included patients with no postbaseline scans available, missing subsets of scans, or patients who discontinued before obtaining adequate scans to assess or confirm response.

Intracranial efficacy was evaluated in patients with BICR-assessed baseline CNS metastases (n = 51). Intracranial ORR was 49% (95% CI: 34.8-63.4), including 8 patients (16%) with an intracranial complete response (CR) and 17 patients (33%) with an intracranial partial response (PR; Table 2). Median intracranial DoR was 12.9 months (95% CI: 7.6-22.5) and median intracranial PFS was 12.0 months (95% CI: 6.7-15.6). Intracranial ORR was similar in patients who had received prior brain radiotherapy (n = 24; intracranial ORR: 50%) and those who had not received any prior brain radiotherapy (n = 27; intracranial ORR: 48.1%).

**Table 2:** Intracranial Efficacy in Patients With Measurable and Nonmeasurable Baseline CNS Metastases by BICR, for the Efficacy-Evaluable Population and the First-Line Population

Efficacy Parameters	Patients With Baseline CNS Metastases (by BICR)	
	Efficacy-Evaluable Population (n = 51)	First-Line Population^a^ (n = 23)
Intracranial objective response, n (%) (95% CI)	25 (49.0) (34.8-63.4)	14 (60.9) (38.5-80.3)
Intracranial best overall response, n (%)		
Complete response	8 (15.7)	3 (13.0)
Partial response	17 (33.3)	11 (47.8)
Stable disease	0	0
Progressive disease	10 (19.6)	2 (8.7)
Non-CR/non-PD	12 (23.5)	6 (26.1)
Missing or unevaluable^b^	4 (7.8)	1 (4.3)
Median intracranial DoR, months (95% CI)	12.9 (7.6-22.5)	12.9 (7.6-22.2)
12-month durable response, % (95% CI)	58.4 (38.7-78.1)	64.3 (39.2-89.4)
18-month durable response, % (95% CI)	41.3 (21.5-61.2)	35.7 (10.6-60.8)
Median intracranial PFS, months (95% CI)	12.0 (6.7-15.6)	15.6 (7.7-21.1)
12-month durable response, % (95% CI)	48.5 (34.4-62.6)	56.5 (36.3-76.8)
18-month durable response, % (95% CI)	28.5 (15.4-41.5)	37.9 (17.7-58.2)

Abbreviations: BICR = blinded independent central review; CI = confidence interval; CNS = central nervous system; CR = complete response; DoR = duration of response; PD = progressive disease; PFS = progression-free survival.

a Patients who had not received any prior lines of systemic therapy in the metastatic setting; exploratory analysis.

b Missing or unevaluable included patients with no postbaseline scans available, missing subsets of scans, or patients who discontinued before obtaining adequate scans to assess or confirm response.

The safety-evaluable population comprised 247 patients, 95% (n = 234) of whom reported ≥1 treatment-related AE adverse event (TRAE). Most frequent TRAEs included dysgeusia (43%), increased weight (38%), and dizziness (35%) (Supplemental Table 2). Most TRAEs were grade 1 to 2 and manageable, and the most frequent grade 3 TRAE was increased weight (n = 28; 11.3%); grade 4 TRAEs were rare (n = 9; 3.6%) and there was 1 death due to a TRAE (dyspnea). TRAEs led to dose interruption, reduction, and discontinuation in 36%, 35%, and 6.9% of patients, respectively. Serious TRAEs were uncommon (n = 35; 14%; Supplemental Table 3).

### Entrectinib in Treatment-Naïve Patients (First-Line Population) With ROS1 Fusion-Positive NSCLC

**Supplemental Figure 1:**
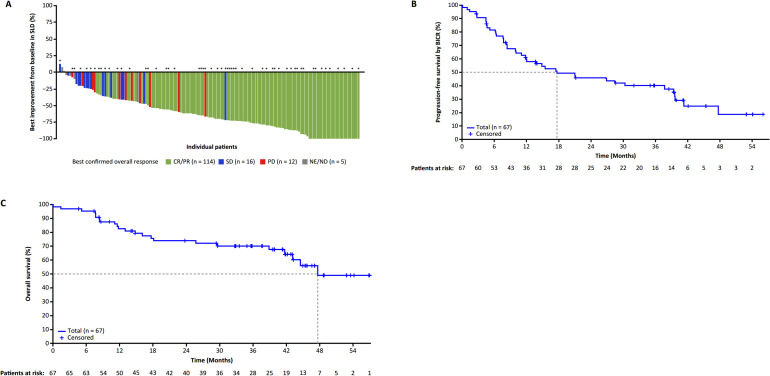
(A) Best overall response in the efficacy-evaluable population and in patients who received entrectinib as first-line treatment (denoted by *), and (B) time-to-event analysis for progression-free survival, and (C) time-to-event analysis for overall survival with entrectinib, in patients with *ROS1* fusion-positive NSCLC who received entrectinib as first-line treatment. Best response was measured at the maximum percentage improvement in the SLD of identified target lesions compared with baseline. Patients with missing SLD change were excluded from the waterfall plot. *Denotes patients who received entrectinib as first-line treatment. Abbreviations: BICR = blinded independent central review; CR = complete response; ND = not determined; NE = not estimable; NSCLC = non–small-cell lung cancer; PD = progressive disease; PR = partial response; *ROS1* = *ROS* proto-oncogene 1; SD = stable disease; SLD = sum of longest diameters.

In the first-line population (n = 67), ORR was 69% (n = 46; 95% CI: 56.2-79.4; Table 1), and most patients had a reduction in the size of their target lesions, similar to the efficacy-evaluable population (Supplemental Figure 1A). Median DoR was 35.6 months (95% CI: 13.9-38.8), median PFS was 17.7 months (95% CI: 11.8-39.4), and median OS was 47.7 months (95% CI: 43.2-not estimable) (Table 1; Supplemental Figure 1B and C). Efficacy endpoints in patients with and without baseline CNS metastases are shown in Table 1. ORR was similar in the 2 groups, but DoR and PFS were longer in patients without baseline CNS metastases versus those with baseline CNS metastases.

Of 23 patients with BICR-assessed baseline CNS metastases (Table 2), 14 (61%; 95% CI: 38.5-80.3) had an intracranial response; 3 (13%) had an intracranial CR, and 11 (48%) had an intracranial PR. Median intracranial DoR was 12.9 months (95% CI: 7.6-22.2), and median intracranial PFS was 15.6 months (95% CI: 7.7-21.1).

Safety data in the first-line safety-evaluable population (n = 87) were consistent with those in the overall safety-evaluable population.

## Discussion

We report updated efficacy and safety data from the integrated analysis of 3 trials of entrectinib in patients with *ROS1* fusion-positive NSCLC, with 4 more patients and longer follow-up than the previous report.^9^ The overall and intracranial efficacy of entrectinib were similar to those reported previously, supporting our prior findings of the activity of entrectinib in this patient population.^9^

We also provide the first report from an exploratory subgroup analysis of patients within these studies who received entrectinib as first-line treatment (first-line population). The ORR in this population was similar to that in the efficacy-evaluable population (69% and 67%, respectively), as were the median PFS and median OS. Responses were more durable in the first-line population with a numerically longer median DoR compared with the efficacy-evaluable population (35.6 vs. 20.4 months, respectively); however, the 95% CIs for the 2 values were wide and overlapping. This observed difference is likely due to patients in the efficacy-evaluable population who were heavily pretreated (23% had received ≥2 prior lines of systemic therapy) and thus typically have a shorter duration of benefit following prior treatments compared with treatment-naïve patients.^10^ As expected, patients without baseline CNS metastases had longer median DoR and median PFS versus those with baseline CNS metastases, in both the efficacy-evaluable and first-line populations.

Our data support current guidelines recommending entrectinib as a first-line treatment option for patients with *ROS1* fusion-positive NSCLC, including those with baseline CNS metastases.^11^ This is also supported by our earlier finding that entrectinib has only modest overall and intracranial efficacy in patients with CNS-only progression following prior crizotinib treatment.^9^ The first-line population analysis was exploratory and included a relatively small number of patients, therefore any conclusions should be interpreted with caution and require further investigation.

Entrectinib maintained consistent safety data in this updated analysis with those reported previously.^7^^,^^8^^,^^9^ The percentage of patients experiencing a TRAE or a serious TRAE was similar to previous reports. One death due to a TRAE has been reported since the previous analysis.

The limitations of this study, as discussed previously,^7^^,^^8^^,^^9^ include the relatively small sample size, the single-arm study design, and the fact that postprogression tissue collection was not mandated. Additionally, the analysis of the first-line population was exploratory and not statistically powered.

In conclusion, entrectinib has demonstrated durable overall and intracranial responses with longer follow-up in patients with *ROS1* fusion-positive NSCLC, with and without baseline CNS metastases, including those who had received it as a first-line treatment. These data support the use of entrectinib as a first-line treatment for patients with *ROS1* fusion-positive NSCLC.

## CRediT authorship contribution statement

**Yun Fan:** Investigation, Writing – review & editing. **Alexander Drilon:** Investigation, Writing – review & editing. **Chao-Hua Chiu:** Investigation, Writing – review & editing. **Herbert H.F. Loong:** Investigation, Writing – review & editing. **Salvatore Siena:** Investigation, Writing – review & editing. **Maciej Krzakowski:** Investigation, Writing – review & editing. **Rafal Dziadziuszko:** Investigation, Writing – review & editing. **Harald Zeuner:** Investigation, Writing – review & editing. **Cloris Xue:** Software, Formal analysis, Data curation, Writing – review & editing. **Matthew G. Krebs:** Investigation, Writing – review & editing.

## Disclosure

**Dr. Fan** reports receiving honoraria from Heng Rui Therapeutics, AstraZeneca, Bristol-Myers Squibb, BeiGene, Pfizer, Boehringer Ingelheim, and Simcere; participated in a Data Safety Monitoring Board/advisory board for F. Hoffmann-La Roche Ltd.

**Dr. Drilon** reports receiving honoraria from or participating in advisory boards for Ignyta/Genentech/Roche, Loxo/Bayer/Lilly, Takeda/Ariad/Millenium, TP Therapeutics, AstraZeneca, Pfizer, Blueprint Medicines, Helsinn, BeiGene, BergenBio, Hengrui Therapeutics, Exelixis, Tyra Biosciences, Verastem, MORE Health, AbbVie, 14ner/Elevation Oncology, ArcherDX, Monopteros, Novartis, EMD Serono, Medendi, Repare RX, Nuvalent, Merus, Chugai Pharmaceutical, Remedica Ltd, mBrace, AXIS, EPG Health, Harborside Nexus, Liberum, RV More, Ology, Amgen, TouchIME, Janssen, Entos, Treeline Bio, Prelude, Applied Pharmaceutical Science, AiCME, I3 Health, MonteRosa, InnoCare, and Boundless Bio; equity in Treeline Bio; associated research paid to institution from Pfizer, Exelixis, GlaxoSmithKlein, Teva, Taiho, and PharmaMar; copyright to Selpercatinib-Osimertinib (filed/pending); research with Foundation Medicine; royalties from Wolters Kluwer; other (food/beverage) with Merck, Puma, Merus, and Boehringer Ingelheim, and CMS honoraria from Medscape, OncLive, PeerVoice, Physicians Education Resources, Targeted Oncology, Research to Practice, Axis, Peerview Institute, Paradigm Medical Communications, WebMD, MJH Life Sciences, Med Learning, Imedex, Answers in CME, Clinical Care Options, EPG Health, JNCC/Harborside, Liberum, Remedica Ltd, and Lungevity.

**Dr. Chiu** reports receiving honoraria from Amgen, AstraZeneca/MedImmune, Boehringer Ingelheim, Bristol-Myers Squibb, Chugai Pharmaceutical, Eli Lilly, Janssen, Merck KGaA, Merck Sharp & Dohme, Novartis, Ono Pharmaceutical, Pfizer, F. Hoffmann-La Roche Ltd, and Takeda; advisory/consultancy for Bristol-Myers Squibb, Eli Lilly, Janssen, Merck KGaA, Merck Sharp & Dohme, Novartis, and F. Hoffmann-La Roche Ltd.

**Dr. Loong** reports receiving funding from Novartis Pharmaceuticals, AbbVie, Bayer, Eisai, Eli Lilly, Guardant Health, Boehringer Ingelheim, Celgene, Illumina, Merck Sereno, Takeda, and George Clinical.

**Dr. Siena** reports participation in advisory boards for Agenus, AstraZeneca, Bayer, Bristol-Myers Squibb, CheckmAb, Daiichi Sankyo, Guardant Health, Menarini, Merck, Novartis, F. Hoffmann-La Roche Ltd/Genentech, and Seattle Genetics.

**Dr. Krzakowski** reports advisory/consultancy for Amgen, Boehringer Ingelheim, Janssen, and F. Hoffmann-La Roche Ltd.

**Dr. Dziadziuszko** reports advisory/consultancy for F. Hoffmann-La Roche Ltd, Foundation Medicine, Pfizer, AstraZeneca, Novartis, Merck Sharp & Dohme, and Karyopharm; honoraria from F. Hoffmann-La Roche Ltd, AstraZeneca, and Amgen; and participation in Data Safety Monitoring Boards/advisory boards for F. Hoffmann-La Roche Ltd, AstraZeneca, Amgen, and Merck Sharp & Dohme.

**Mr. Zeuner and Ms. Xue** are employees of F. Hoffman-La Roche Ltd.

**Dr. Krebs** reports receiving honoraria from F. Hoffmann-La Roche Ltd; consulting/advisory fees from F. Hoffmann-La Roche Ltd, Guardant Health, Janssen, Seattle Genetics, OM Pharmaceutical Industries, and Bayer; speakers’ bureau for F. Hoffmann-La Roche Ltd, Janssen, and AstraZeneca; research funding from F. Hoffmann-La Roche Ltd, BerGenBio and Novartis; and travel/accommodation/expenses from AstraZeneca, BerGenBio, Immutep, and Janssen.

## Figures and Tables

**Supplemental Table 1:** Baseline Demographics and Disease Characteristics of Patients With *ROS1* Fusion-Positive NSCLC Who Were ROS1 TKI-Naïve (Efficacy-Evaluable Population), and in a Subset of Patients Who Received Entrectinib as First-Line Treatment

Characteristic		Efficacy-Evaluable Population (N = 172)	First-Line Population^a^ (n = 67)
Age, years	Median (range)	54.5 (20-86)	55.0 (33-86)
Sex, n (%)	Male	59 (34.3)	26 (38.8)
	Female	113 (65.7)	41 (61.2)
Race, n (%)	Asian	82 (47.7)	27 (40.3)
	White	72 (41.9)	29 (43.3)
	Black or African American	8 (4.7)	4 (6.0)
	Other or not reported	10 (5.8)	7 (10.4)
ECOG PS, n (%)	0	66 (38.4)	25 (37.3)
	1	90 (52.3)	37 (55.2)
	2	16 (9.3)	5 (7.5)
Smoking status, n (%)	Never smoker	111 (64.5)	42 (62.7)
	Former or current smoker	61 (35.5)	25 (37.3)
Histology, n (%)	Adenocarcinoma	166 (96.5)	67 (100.0)
	Adenosquamous carcinoma	1 (0.6)	0
	Bronchioloalveolar carcinoma	1 (0.6)	0
	NSCLC – not otherwise specified	4 (2.3)	0
Prior lines of systemic therapy, n (%)^b^	0	67 (39.0)	67 (100.0)
	1	65 (37.8)	N/A
	≥2	40 (23.3)	N/A
CNS metastases at baseline, n (%)^c^	Yes	60 (34.9)	26 (38.8)
	No	112 (65.1)	41 (61.2)
Prior radiotherapy of the brain, n (%)^d^	Yes	27 (45.0)	11 (42.3)
	No	33 (55.0)	15 (57.7)
Any prior therapy, n (%)	Chemotherapy	115 (66.9)	13 (19.4)
	Immunotherapy	27 (15.7)	2 (3.0)
	Targeted therapy	14 (8.1)	0
	Hormonal Therapy	1 (0.6)	1 (1.5)
*ROS1* fusion partner, n (%)	CD74	73 (42.4)	29 (43.3)
	EZR	25 (14.5)	9 (13.4)
	SLC34A2	22 (12.8)	8 (11.9)
	SDC4	14 (8.1)	6 (9.0)
	TPM3	3 (1.7)	1 (1.5)
	ZCCHC8	2 (1.2)	1 (1.5)
	CCDC6	1 (0.6)	1 (1.5)
	KDELR2	1 (0.6)	0
	LRIG3	1 (0.6)	1 (1.5)
	MSN	1 (0.6)	0
	MVP	1 (0.6)	1 (1.5)
	MYH9	1 (0.6)	0
	PWWP2A	1 (0.6)	1 (1.5)
	WNK1	1 (0.6)	1 (1.5)
	Undetected	1 (0.6)	1 (1.5)
	Unknown	24 (14.0)	7 (10.4)

Abbreviations: CNS = central nervous system; ECOG PS = Eastern Cooperative Oncology Group performance status; N/A = not applicable; NSCLC = non–small-cell lung cancer; *ROS1* = *ROS* proto-oncogene 1; TKI = tyrosine kinase inhibitor.

a Patients who had not received any prior lines of systemic therapy in the metastatic setting; exploratory analysis.

b Lines of therapy determined from the time of metastatic disease diagnosis. Patients may have received other therapies in the adjuvant or neo-adjuvant setting.

c CNS metastases at baseline as judged by the investigator.

d In patients with baseline CNS metastases as judged by the investigator.

**Supplemental Table 2:** Treatment-Related Adverse Events in the *ROS1* Fusion-Positive NSCLC Overall Safety-Evaluable Population and First-Line Safety-Evaluable Population

TRAEs (≥10% of Patients)	Overall Safety-Evaluable Population (N = 247)	First-Line Safety-Evaluable Population^a^ (n = 87)
Total number of patients with ≥1 event	234 (94.7)	85 (97.7)
Dysgeusia	105 (42.5)	51 (58.6)
Increased weight	93 (37.7)	40 (46.0)
Dizziness	87 (35.2)	32 (36.8)
Constipation	80 (32.4)	28 (32.2)
Diarrhea	75 (30.4)	25 (28.7)
Fatigue	66 (26.7)	22 (25.3)
Blood creatinine increased	61 (24.7)	28 (32.2)
Oedema peripheral	53 (21.5)	20 (23.0)
Nausea	49 (19.8)	16 (18.4)
Paresthesia	47 (19.0)	16 (18.4)
Anemia	40 (16.2)	18 (20.7)
Myalgia	40 (16.2)	12 (13.8)
Vomiting	38 (15.4)	10 (11.5)
Gait disturbance	16 (6.5)	10 (11.5)
Aspartate aminotransferase increased	36 (14.6)	15 (17.2)
Alanine aminotransferase increased	34 (13.8)	15 (17.2)
Arthralgia	30 (12.1)	11 (12.6)
Dysphagia	27 (10.9)	12 (13.8)
Hyperuricemia	27 (10.9)	9 (10.3)
Rash	21 (8.5)	9 (10.3)
Hypotension	14 (5.7)	9 (10.3)

Abbreviations: TRAE = treatment-related adverse event; NSCLC = non–small-cell lung cancer; *ROS1* = *ROS* proto-oncogene 1.

a Patients who had not received any prior lines of systemic therapy in the metastatic setting; exploratory analysis.

**Supplemental Table 3:** Treatment-related serious AEs in the *ROS1* fusion-positive NSCLC overall safety-evaluable population and first-line safety-evaluable population

Treatment-Related Serious AEs	Overall Safety-Evaluable Population (N = 247)	First-Line Safety-Evaluable Population^a^ (n = 87)
Total number of patients with ≥1 serious AE	35 (14.2)	17 (19.5)
Pyrexia	4 (1.6)	–
Cardiac failure	3 (1.2)	2 (2.3)
Vomiting	2 (0.8)	1 (1.1)
Congestive cardiac failure	2 (0.8)	2 (2.3)
Cognitive disorder	2 (0.8)	–
Ataxia	1 (0.4)	1 (1.1)
Dysarthria	1 (0.4)	–
Limbic encephalitis	1 (0.4)	1 (1.1)
Acute coronary syndrome	1 (0.4)	–
Myocarditis	1 (0.4)	–
Sinus arrhythmia	1 (0.4)	–
Dehydration	1 (0.4)	1 (1.1)
Hyperkalemia	1 (0.4)	1 (1.1)
Hypertriglyceridemia	1 (0.4)	1 (1.1)
Hypervolemia	1 (0.4)	–
Anorectal disorder	1 (0.4)	–
Diarrhea	1 (0.4)	–
Muscular weakness	1 (0.4)	1 (1.1)
Osteoarthritis	1 (0.4)	–
Intervertebral disc protrusion	1 (0.4)	–
Spinal stenosis	1 (0.4)	1 (1.1)
Mental status changes	1 (0.4)	1 (1.1)
Dyspnea	1 (0.4)	1 (1.1)
Delirium	1 (0.4)	1 (1.1)
Hypotension	1 (0.4)	1 (1.1)
Orthostatic hypotension	1 (0.4)	1 (1.1)
Blood creatinine increased	1 (0.4)	–
Urinary tract infection	1 (0.4)	1 (1.1)
Tendon rupture	1 (0.4)	–
Acute kidney injury	1 (0.4)	1 (1.1)
Rash	1 (0.4)	–

Abbreviations: AE = adverse event; NSCLC = non–small-cell lung cancer; *ROS1* = *ROS* proto-oncogene 1.

a Patients who had not received any prior lines of systemic therapy in the metastatic setting; exploratory analysis.

## Supplementary Material

### Safety Assessment

Adverse events (AEs) were graded according to the National Cancer Institute Common Terminology Criteria for AEs (v4.03) and coded using the Medical Dictionary for Regulatory Activities dictionary (v24.0). If necessary, ≤2 dose reductions in 200 mg decrements were permitted.

### Statistical Analyses

Continuous data were summarized using means, standard deviations, medians, and ranges. Categorical data were summarized by counts and proportions. The number, proportion, and the corresponding 2-sided 95% Clopper-Pearson exact confidence intervals (CI) were summarized for ORR. The median of time-to-event endpoints (DOR, PFS, OS) were estimated using the Kaplan-Meier method. The associated 2-sided 95% CIs were calculated using the method of Brookmeyer and Crowly. Landmark analyses were provided with the corresponding 2-sided 95% CIs calculated using the method of Kalbfleisch and Prentice.
